# The Evolution of Physical Performance throughout an Entire Season in Female Football Players

**DOI:** 10.3390/sports12020052

**Published:** 2024-02-06

**Authors:** Francisco Reyes-Laredo, Fernando Pareja-Blanco, Guillermo López-Lluch, Elisabet Rodríguez-Bies

**Affiliations:** 1 Department of Physiology, Anatomy and Cell Biology, Andalusian Centre of Developmental Biology, Universidad Pablo de Olavide, 41013 Seville, Spain; freylar@acu.upo.es (F.R.-L.); glopllu@upo.es (G.L.-L.); 2 Physical Performance & Sports Research Center, (CIRFD), Universidad Pablo de Olavide, 41013 Seville, Spain; fparbla@upo.es; 3 Department of Sport and Computer Sciences, Faculty of Sport Sciences, Universidad Pablo de Olavide, 41013 Seville, Spain; 4 Centro de Investigación Biomédica en Red de Enfermedades Raras (CIBERER, U729), Instituto de Salud Carlos III, 28029 Madrid, Spain

**Keywords:** seasonal training effects, female soccer player adaptation, jump, sprint, change of direction, strength

## Abstract

Research on the evolution of performance throughout a season in team sports is scarce and mainly focused on men’s teams. Our aim in this study was to examine the seasonal variations in relevant indices of physical performance in female football players. Twenty-seven female football players were assessed at week 2 of the season (preseason, PS), week 7 (end of preseason, EP), week 24 (half-season, HS), and week 38 (end of season, ES). Similar to the most common used conditioning tests in football, testing sessions consisted of (1) vertical countermovement jump (CMJ); (2) 20 m running sprint (T20); (3) 25 m side-step cutting maneuver test (V-CUT); and (4) progressive loading test in the full-squat exercise (V1-LOAD). Participants followed their normal football training procedure, which consisted of three weekly training sessions and an official match, without any type of intervention. No significant time effects were observed for CMJ height (*p* = 0.29) and T20 (*p* = 0.11) throughout the season. However, significant time effects were found for V-CUT (*p* = 0.004) and V1-LOAD (*p* = 0.001). V-CUT performance significantly improved from HS to ES (*p* = 0.001). Significant increases were observed for V1-LOAD throughout the season: PS-HS (*p* = 0.009); PS-ES (*p* < 0.001); EP-ES (*p* < 0.001); and HS-ES (*p* = 0.009). These findings suggest that, over the course of the season, female football players experience an enhancement in muscle strength and change of direction ability. However, no discernible improvements were noted in sprinting and jumping capabilities during the same period.

## 1. Introduction

Football is one of the most popular sports worldwide. Up to 265 million people practice it [1]. According to the last report of the Fédération Internationale de Football Association, more than 13.3 million women players are officially registered [2]. Despite the growing popularity of women’s football, there is still limited scientific research focused on female players in comparison with their male counterparts, especially about the physical characteristics and physiological demands related to specific football practice [3].

Men football players cover, on average, about 10 km of distance per game, with high intensity runs of 2 to 3 s (120–150 times per game) [4,5]. On the other hand, women football players cover a total distance between 4 and 13 km per game, from which 0.2–1.7 km are covered at high-speed [6]. Although high-intensity actions in matches only account for 8% to 12% of the distance covered, they are considered important for performance capabilities [7]. Indeed, the high-speed distance undertaken by female players increases at higher standard levels of competition [8].

High physical fitness allows players to deal with football game demands [6]. Female football players show 20 m sprint times ranging from 3.05 to 3.59 s [9,10,11] and vertical countermovement jump (CMJ) heights between 28 and 43 cm [6]. In this regard, sprint and jump performances may vary according to players’ standard level. For instance, Haugen et al. (2012) reported that international female football players are 2–5% faster over 20 m than their national-level counterparts [10]. Moreover, international female football players jump 8–9% higher than national players [10]. Likewise, the ability to rapidly enact change of direction (COD) has been suggested as a principal fitness parameter, due to the frequent and explosive directional changes performed by football players during the game [12]. COD studies have demonstrated that male players perform faster CODs than female players but only 7.5% faster [13]. Likewise, these authors did not find differences between men and women football players in the “Functional Movement Test”, indicating that, in football, some differences between male and female groups are minor [13].

In line with sprint and jump performance, it has also been shown that COD performance is related to competitive standard [14]. Sprinting, jumping, and cutting abilities may be dependent on the ability to apply force [15]. A recent study has shown that starting female football players attain greater strength-based performance than non-starters [16]. These findings support the idea that fitness performance should be assessed to individualize training programs according to their current fitness level and competitive standard.

Previous studies conducted with professional male football players have not shown any significant changes in jump height throughout the season [17]. Others found that male football players improved jump and sprint performances from preseason to midseason, but no further performance gains were observed from midseason onward [18,19]. However, it has been reported that professional male football players achieved their best sprint performance at the end of the competitive period [20]. Likewise, other authors have reported significant declines in aerobic capacity and vertical jump from the preseason until the end of the season, although only two time points were measured [21]. A recent study conducted with elite female Portuguese football players did not find any improvement in physical performance following a 5-week training intervention [22].

To our knowledge, no study has assessed seasonal variation in high-intensity performance indicators in female football players. Since men and women show different physiological profiles [22], male and female football players may not present similar behaviors throughout the season. For instance, a greater proportion of type I fibers in specific muscle groups among females [23] may influence program design and subsequent adaptations for men and women. Knowledge of physical performance during the season may provide coaches valuable information about the orientation of the strength and conditioning training program that should be conducted in each season phase. Therefore, the aim of this study was to examine the changes in relevant indices of physical performance throughout a season in female football players. Based on the previous literature, the hypothesis of our study is that physical performance will improve during the preseason, but no further improvements will be observed from the midseason.

## 2. Materials and Methods

### 2.1. Subjects

Thirty-one female football players from two teams competing in the regional championship were initially recruited and voluntarily participated in this investigation. Due to injuries, four subjects could not perform all the testing sessions and dropped out of the study. Thus, the final dataset was obtained from the remaining 27 (20 from one team and 7 from the other) participants (21.2 ± 7.6 years, height 1.61 ± 0.07 m, and body mass 61.4 ± 9.6 kg). Participants were homogeneous regarding skill levels since they competed in the same category. The subjects were informed about the research procedures and gave their written informed consent to participate. In the case of these participants aged less than 18 years, informed consent was obtained from their parents/guardians. No physical limitations, health problems, or injuries were found that could affect the tests. None of the participants were using drugs or medications known to influence physical performance. All the procedures performed in this study were approved by the Ethics Committee for Biomedical Research of the Andalusian Government with the number 2355-N-19, following the indications of the International Conference of Good Clinical Practices, and were conducted in accordance with the 1975 Declaration of Helsinki guidelines.

### 2.2. Experimental Approach to the Problem

This investigation was performed over 38 weeks (about 9 months), and it was split into 4 periods: preseason (PS), end of preseason (EP), half-season (HS), and end of season (ES). This is a diagnostic study to identify the normal evolution of players in a normal training context. To avoid disturbances in the training procedure, participants carried out their scheduled football training protocol for the regional league category, which generally consisted of three weekly training sessions and an official match, without any type of intervention from the researchers. During the 90 min training sessions, technical, tactical, and conditioning football aspects were worked on (Table 1). This program was essentially maintained throughout the season with minor modifications of the percentages at each specific phase. On Tuesday, Thursday, and Friday, some strength exercises were conducted focused on injury prevention. On Tuesday, 2–3 sets of 8–10 repetitions of squats and deadlifts were performed with a Russian belt (without external load). Moreover, about 20 unilateral and bilateral jumps were performed. On Thursday and Friday, some accelerations and COD tasks were conducted as follows: 6–10 repetitions of 10–20 m with 30–60 s rest. During the study, the players did not perform any other sport or specific conditioning training.

Physical performance was examined in week 2 for PS, week 7 for EP, week 24 for HS, and week 38 for ES. Testing sessions consisted of (1) vertical countermovement jump (CMJ); (2) 20 m running sprint (T20); (3) 25 m side-step cutting maneuver test (V-CUT); and (4) progressive loading test in the full-squat exercise (V1-LOAD). Testing sessions were carried out at least 48 h after the most recent game. All the measurements were performed at the same time of the day for each player and under controlled environmental conditions (20–22 °C and 55–65% humidity) in a research laboratory. Subjects underwent a familiarization session 2 weeks before the start of the first trial. These sessions were supervised by researchers. Attention was paid to ensuring that proper exercise techniques were used, and detailed instruction was provided for the specific testing procedures.

### 2.3. Procedures

Before measurements, a 15 min warm-up was performed, which consisted of 5 min of jogging, joint mobility, and a specific warm-up for every test. During all measurements, participants were encouraged to give their best performance, and performance feedback was given after each trial.

#### 2.3.1. Vertical Countermovement Jump (CMJ)

Jump height was calculated at the nearest 0.1 cm from flight time measured with an infrared timing system (OptojumpNext, Microgate, Bolzano, Italy). Because the take-off and landing positions can affect the jump flight, strict instructions were addressed to all participants to keep their legs straight during the flight time of the jump. The subjects started from an upright standing position, made a downward movement until approximating a knee angle of 90°, and subsequently began to push off at maximal velocity. All participants completed 5 maximal CMJs, with their hands on their hips, separated by 1 min rest. The highest and the lowest values were discarded, and the resulting average value was kept for analysis. The specific warm-up consisted of two sets of 10 squats without external load, 5 submaximal CMJs, and 3 maximal CMJs. The test–retest reliability was as follows: intraclass correlation coefficient (ICC) was 0.93 (95% confidence interval (CI): 0.86; 0.97), and the coefficient of variation (CV) was 1.7%.

#### 2.3.2. Running Sprints (T20)

Two 20 m straight-line sprints, separated by a 3 min rest interval, were performed on an indoor running track. The fastest sprint time was chosen for further analysis (T20). Photocell timing gates (Witty, Microgate, Bolzano, Italy) were placed at 0 and 20 m. A standing start with the leadoff foot placed 1 m behind the first timing gate was used. The warm-up protocol comprised four 20 m running accelerations at 80%, 85%, 90%, and 95% of the perceived effort and one 10 m sprint at 100% effort with 1 min rest between them. The reliability, measured by ICC (95% CI) and CV was 0.99 (0.98; 1.00) and 2.8%, respectively.

#### 2.3.3. Twenty-Five m Side-Step Cutting Maneuver Test (V-CUT) 

In the V-CUT test, the subjects performed a 25 m sprint with 4 CODs of 135° every 5 m. For the test to be valid, the subjects had to pass the line, drawn on the ground, with one foot completely in each turn. If the trial was deemed unsuccessful, a new trial was allowed. The distance between each pair of cones was 0.7 m. Subjects performed 2 tests, separated by a 3 min rest interval. The fastest time was saved for further analysis. Photocell timing gates (Witty, Microgate, Bolzano, Italy) were placed at the beginning and the end of the trial. A standing start was used with the starting foot positioned 1 m behind the first timing gate. The warm-up protocol consisted of one submaximal trial. The ICC was 0.95 (95% CI: 0.88; 0.98), and the CV was 1.8%.

#### 2.3.4. Progressive Loading Test in the Full-Squat Exercise (V1-LOAD) 

The assessment consisted of increasing loads using the full-squat exercise on a Smith machine (Multipower Fitness Line, Peroga, Murcia, Spain). The full-squat exercise was performed with subjects starting from the upright position with the knees and hips fully extended, stance approximately shoulder width apart, and the barbell resting across the back at the level of the acromion. Each participant descended in a continuous motion until the top of the thighs reached below the horizontal plane, then immediately reversed motion, and ascended back to the upright position. Feedback based on eccentric distance traveled and concentric velocity was provided. This was accomplished by using a linear velocity transducer (T-Force System, Ergotech, Murcia, Spain) that registered the kinematics of every repetition at 1000 Hz and whose software provided visual and auditory feedback in real time. Unlike the eccentric phase that was performed at a controlled mean velocity (i.e., 0.50–0.65 m·s^−1^), athletes were required to execute the concentric phase at maximal velocity. The initial load was set at 20 kg and was progressively increased in 10 kg increments until the mean propulsive velocity (MPV) was 1.00 m·s^−1^ (range: 0.95–1.05 m·s^−1^). The load that each subject could lift at 1.00 m·s^−1^ (V1LOAD) was chosen for further analysis. Subjects performed 3 repetitions with each load. Four-minute rests were taken between sets. The warm-up consisted of one set of 6 repetitions (3 min rest) with 20 kg. MPV corresponds to the mean velocity of the propulsive phase of each repetition. The propulsive phase was defined as that portion of the concentric phase during which the measured acceleration is greater than the acceleration due to gravity (i.e., <−9.8 m·s^−2^) [24]. For the MPV attained against 20 kg, the ICC was 0.99 (95% CI: 0.97; 0.99), and the CV was 2.4%.

### 2.4. Statistical Analyses

The values are reported as mean ± standard deviation (SD). The reliability of the measures was calculated via the ICC with 95% CI, using the one-factor random model, and the CV. Normality of the data and homoscedasticity were verified using the Shapiro–Wilk and Levene tests, respectively. One-way repeated ANOVA measures was used to detect differences between the different time points in the assessed variables. Bonferroni’s post hoc adjustments were applied for all pairwise multiple comparisons. Associations between variables at PS were examined by Pearson’s correlation analysis. Statistical significance was established at the level of *p* ≤ 0.05. Statistical analysis was carried out using SPSS version 25 for Windows (IBM Corp., Armonk, NY, USA).

To determine the effect sizes, Cohen’s *d_s_* was calculated as the mean value of trial 2 minus mean value trial 1 divided by the pooled SD [25]. The effect size was classified regardless of the sign as indicated by Sawilowsky [26] as follows: d(0.01) = very small, d(0.2) = small, d(0.5) = medium, d(0.8) = large, d(1.2) = very large, and d(2.0) = huge. Sample size was calculated (using GPower Version 3.1.9.2) introducing the following parameters: *effect size* 0.3 (low, based on previous literature); α error probability (0.05); and power (0.95), with only one group and 4 measurements, which resulted in a sample size of 27 subjects.

## 3. Results

The descriptive characteristics of physical performance in female football players throughout the different phases of the season (PS, EP, HS, ES) are reported in Table 2.

**Figure 1:**
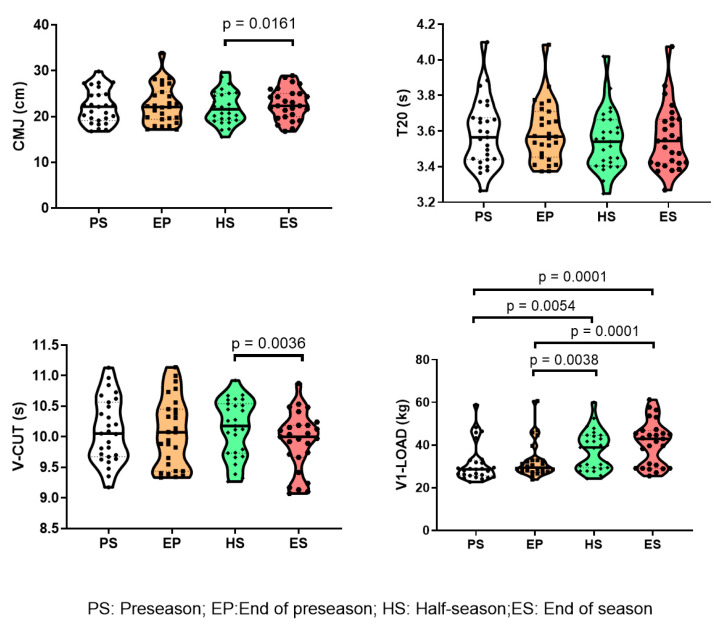
Individual evolution of physical performance throughout the season. The data represent the distribution with the median (line) of the whole population throughout different phases of the football season (n = 27). CMJ: height attained in the vertical countermovement jump (cm); T20: time in the 20 m running sprint; V-CUT: time in the V-CUT test, which was conducted to assess the change of direction performance; V1-LOAD: absolute load (kg) lifted at a mean propulsive velocity of 1.00 m·s^−1^ in the full-squat exercise. Preseason (PS), end of preseason (EP), half-season (HS), and end of season (ES). Individual scores are indicated. The line represents the median of the population. The dotted line represents quartiles. Significant differences between different phases of the season are indicated.

No significant time effects were observed for CMJ height (*p* = 0.29) and T20 (*p* = 0.11) throughout the season. By contrast, significant time effects were found for V-CUT (*p* = 0.004) and V1-LOAD (*p* = 0.001). V-CUT performance significantly improved from HS to ES (*p* = 0.001). Significant increases were observed for V1-LOAD between almost all measurements throughout the season, indicated as follows: PS-HS (*p* = 0.009), PS-ES (*p* < 0.001); EP-ES (*p* < 0.001); HS-ES (*p* = 0.009). Figure 1 depicts individual responses to the different physical performance indicators assessed throughout the season.

Interestingly, throughout the season, the V-CUT score clearly condensed around the mean of the score of the population in PS, whereas the V1-LOAD clearly evolved from a low score in PS to a wider distribution at the end of the season in which three different populations can be perceived: a population that is maintained at the lower score, a numerous population in the center of the distribution, and some participants that show a high score (Figure 1).

Effect size values, calculated as Cohen’s d, are described in Table 3.

**Figure 2:**
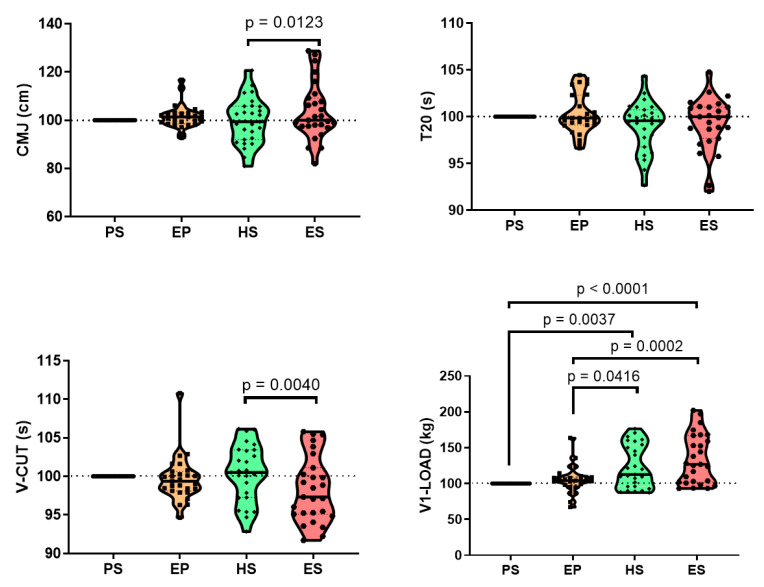
Percentage of change in physical performance throughout the season. The data represent the distribution of the percentage of change vs. PS score of the whole population throughout different phases of the football season (n = 27). CMJ: height attained in vertical countermovement jump (cm); T20: time in 20 m running sprint; V-CUT: time in the V-CUT test, which was conducted to assess the change of direction performance; V1-LOAD: absolute load (kg) lifted at a mean propulsive velocity of 1.00 m·s^−1^ in the full-squat exercise. Preseason (PS), end of preseason (EP), half-season (HS), and end of season (ES). Individual scores are indicated. The line represents the median of the population. The dotted line represents quartiles. Significant differences between different phases of the season are indicated.

When we determined the percentage of change in physical performance of the participants in the study, we found that only in V-CUT and V1-LOAD, a significant change was found. In the case of V-CUT, most of the participants showed a reduction in the score in comparison with the score in PS indicating a better performance. On the other hand, in V1-LOAD most of the population increase the score reading a 100% of increase in comparison with the score at PS. Although not significantly, T20 also showed a tendency to decrease in the score in most of the participants. And in CMJ, the tendency was to increase, specially at the end of the season (Figure 2).

**Figure 3:**
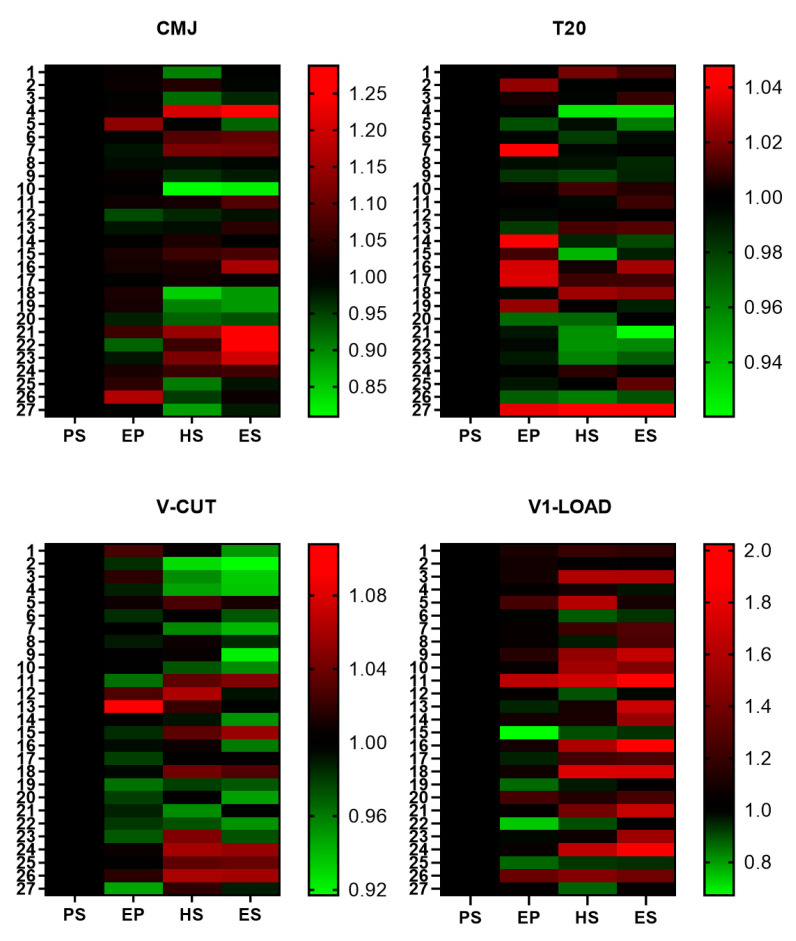
Heatmap of the change in physical performance throughout the season. The data represent the individual changes vs. PS score of the whole population throughout different phases of the football season (n = 27). CMJ: height attained in the vertical countermovement jump (cm); T20: time in the 20 m running sprint; V-CUT: time in the V-CUT test, which was conducted to assess the change of direction performance; V1-LOAD: absolute load (kg) lifted at a mean propulsive velocity of 1.00 m·s^−1^ in the full-squat exercise. Preseason (PS), end of preseason (EP), half-season (HS), and end of season (ES). Individual score at PS was considered as reference and indicated in black.

To better visualize this progression, we performed heatmaps of the distribution of the different scores individually as shown in Figure 3. Taking as reference the scores at the beginning of the season (considered with a value of 1 = black), most of the participants in this study performed better in the V-CUT and clearly in the test of V1-LOAD. In the case of the T20 test, the differences throughout the season were very low, and, interestingly, a better performance was detected in many of the participants in the beginning of the championship (EP), but the performance worsened throughout the season. In the case of the CMJ test, the changes throughout the season were more aleatory.

When we correlated the different parameters, significant relationships were observed between CMJ height and T20 (r = −0.66, *p* < 0.001) and between V-CUT and V1-LOAD (r = −0.39; *p* < 0.05). No significant relationships were observed between the other parameters assessed (Table 4).

## 4. Discussion

To our knowledge, this is the first study showing the evolution of physical performance throughout an entire season in female football players. Most of the studies carried out so far are based on punctual determinations at one moment throughout a season [19,22,27]. Our study comprises a whole season of a total of 27 players from two teams playing in the same football category. Our results indicate that specific football training may not be enough to improve jumping and sprinting capabilities during the season in female football players, although it seems to have a positive impact on COD performance and muscle strength, especially in the last stage (ES). Strength and conditioning coaches should know the physical performance of their players throughout the season to design proper training programs.

Jump performance, specifically CMJ, is an accepted indicator of lower body power production in football players [28]. Furthermore, it is considered a discriminating variable in male football players of different competitive standards [29,30]. Our players jumped about 22 cm, and they did not improve too much during the season. These jump heights do not attain the threshold suggested in previous studies [30] for elite female football players—between 28 and 43 cm. In line with our results, previous studies also observed no changes in jump height during a season [17].

Like jump ability, sprint performance in our participants did not change throughout the season. Other authors reported some improvements in jump height and sprint times in the early season (from preseason to midseason) period but no further changes in the second half of the season in professional male football players [18,19,31]. Football players involved in this study ran 20 m in ~3.55 s, which is close to the lowest limit reported in the literature for female football—3.05–3.59 s [9,10,11]. It has been shown that 20 m sprint times in international female football players is about 3.30 s [32]. The fact that vertical jump and sprint performance show similar behavior is not strange. Sprint performance and CMJ height have shown strong relationships [33], and both have been correlated with the performance in championships throughout the season in professional women footballers [34]. Furthermore, significant associations between CMJ height and sprint performance in various sprint distances in female football and lacrosse players (r = 0.53 to 0.77) have been reported [35]. These results are in line with the relationships observed in this study between CMJ height and T20 (r = −0.66, *p* < 0.001, Table 3). Thus, jumping and sprinting seem to share some underlying mechanisms that explain similar behaviors in both tests. A potential explanation for the lack of improvement in jumping and sprinting performance might be related to interfering adaptations resulting from the high volume of endurance training accumulated in the football sessions [27]. In agreement with our results, a recent study performed on 28 women footballers of one international team observed during four different international tournaments demonstrated that the accumulation of time on the pitch negatively affects performance [36]. Therefore, due to the relevance of these fitness parameters in football performance and the lack of improvement in them throughout the season, strength and conditioning coaches should consider specific fitness programs targeting the improvement of jumping and sprinting capabilities when dealing with female football players.

Contrary to jumping and sprinting capabilities, COD and muscle strength performances increased throughout the season, especially in the last stage (ES). COD is determined by muscle strength, coordination, and flexibility [37]. Moreover, COD performance is also explained by motor control and technical gestures [38]. Specific football practice involves multiple high-intensity COD, along with technical and tactical elements of the game, also stressing cognitive functions [12,37], which may act as COD training. However, these improvements would likely be even greater if a specific COD training program is conducted. Likewise, the increases in muscle strength may explain the improvements in COD performance. Indeed, this suggestion is supported by the significant relationship observed between V-CUT and V1-LOAD (r = −0.39; *p* < 0.05, Table 3). In this regard, some authors highlight the importance of improving leg strength in female football players to increase COD performance [39,40]. However, the scarce number of studies in female football players hampers the direct comparison of our strength findings. The increases in COD performance during the season are partially in line with previous studies [18,31] that reported improvements in COD performance in the preseason in professional male football players, but no further increases were observed during the rest of the season. Notwithstanding, other authors have also observed improvements in COD performance for young elite male football players throughout the entire season (preseason and competitive season) [41]. The changes in training volume and intensity over the distinct season stages may explain the evolution of the different capabilities throughout the season. Indeed, it may be argued that the decrease in aerobic training throughout the season may help to increase explosive performance at the end of the season; however, the cumulative stress of the season may attenuate explosive performance-related adaptations. Unfortunately, representing a study limitation, the training load could not be monitored.

Despite the relevance of muscle strength to general and specific sports skills having been emphasized [42], research on muscle strength in female football players is limited. In this regard, traditional strength tests (e.g., one-repetition maximum (1-RM)) present important shortcomings, mainly when they are used with non-resistance-trained subjects, such as (1) very time-consuming and laborious procedures; (2) a high risk of injury when performed incorrectly; (3) potentially inducing high levels of muscle damage, potentially hampering physical and technical performance on the following days [43]; and (4) the fact that the value measured may not represent the athletes’ actual performance, since they are not familiarized with such loads. Consequently, faster and safer practical methods must be implemented to assess muscle strength in this population. In this regard, the greater the velocity against a given load, the higher the applied force indicating greater strength performance, while a decrease in bar velocity would suggest an impaired ability to apply force [44]. In this regard, female football players increased their leg strength throughout the season. As mentioned above, these strength improvements may be partially responsible for the observed gains in COD ability. However, it seems that specific sprint and plyometric training should be implemented aiming to increase performance in these explosive skills.

One of the limitations of this study is the lack of differentiation between players depending on their positional role in the field. To obtain good information and enough statistical power to reach a conclusion, a pool of players from different teams is needed, and this is very complex because the availability of players and teams for these studies is limited. If we consider professional teams in which the positions are more stable than in other teams, the complexity is even greater. As indicated by other researchers, the position demands can affect even the training procedure for each player [45]. Furthermore, our participants are non-professional players that participate in a regional championship. A comparison with professional players in a professional league would be interesting, but the unavailability of these players made the comparison impossible. Future studies considering these players would clarify the behavior of these and other parameters in more stressful competitions.

For future studies, more determinations such as max VO_2_max and body composition will be useful to obtain a more complete picture of the participants and their capacities.

## 5. Conclusions

In our group of female football players, their normal training program improved their muscle strength and COD performance throughout the season, suggesting an enhancement in muscle strength and COD ability. However, no significant changes were observed in jumping and sprinting capabilities. These findings may be the result of high-intensity accelerations, decelerations, and COD involved in specific football training but also the volume of endurance-type training typically accumulated in these sessions. Whether these findings are generalized to all standard levels should be further investigated. Based on these findings, systematic strength and speed training is required in this population. Future research should investigate the role of different training types on performance outcomes throughout a season in female football players.

These findings provide relevant information about the seasonal evolution of physical performance in female football players. The physical capacities of female football players should be tested regularly during the season to (1) assess the effect of a specific season stage, (2) measure individual and team fitness standards, and (3) design individualized training programs based on athletes’ requirements.

## Figures and Tables

**Table 1:** Weekly training scheme over the 38 weeks.

Sunday	Monday	Tuesday	Wednesday	Thursday	Friday	Saturday
Match	Rest	30% ST 40% TT 30% SG	Rest	10% ST 10% Te 30% TT 20% SG 30% ET	10% ST 10% Te 40% TT 20% SG 20% ET	Rest

Strength training (ST); Tactical training (TT); Small-sided games (SG); Technical training (Te); Endurance training (ET).

**Table 2:** Changes in fitness variables of female football players (n = 27) during the four stages: preseason (PS), end of preseason (EP), half-season (HS), and end of season (ES).

Parameter/Stage	PS	EP	HS	ES	*p*-Value Time Effect
CMJ (cm)	22.2 ± 3.7	22.6 ± 4.2	22.0 ± 3.6	22.8 ± 3.3	0.29
T20 (s)	3.56 ± 0.19	3.56 ± 0.17	3.51 ± 0.17	3.53 ± 0.18	0.11
V-CUT (s)	9.98 ± 0.50	9.96 ± 0.53	10.07 ± 0.47	9.81 ± 0.47 ^HS^	0.004
V1-LOAD (kg)	31.5 ± 9.0	32.5 ± 8.2	37.6 ± 9.2 ^PS^	40.9 ± 10.3 ^PS, EP, HS^	0.001

Data are expressed as mean ± standard deviation. CMJ: height attained in the vertical countermovement jump; T20: time in the 20 m running sprint; V-CUT: time in the V-CUT test, which was conducted to assess the change of direction performance; V1-LOAD: absolute load (kg) lifted at a mean propulsive velocity of 1.00 m·s^−1^ in the full-squat exercise. ^PS^ Significant differences with PS at the corresponding time point (*p* < 0.05). ^EP^ Significant differences with EP at the corresponding time point (*p* < 0.05). ^HS^ Significant differences with HS at the corresponding time point (*p* < 0.05).

**Table 3:** Effect sizes (Cohen’s *d*s) of the comparison of the different variables in female football players (n = 27) during the four stages: preseason (PS), end of preseason (EP), half-season (HS), and end of season (ES).

Parameter	Stage	PS	EP	HS
**CMJ**	**PS**	-		
	**EP**	0.098	-	
	**HS**	0.032	0.143	-
	**ES**	0.098	0.072	0.243
**T20**	**PS**	-		
	**EP**	0.346	-	
	**HS**	1.620	2.102	-
	**ES**	1.079	1.508	0.526
**V-CUT**	**PS**	-		
	**EP**	0.495	-	
	**HS**	0.520	1.015	-
	**ES**	2.610	1.983	3.294
**V1-LOAD**	**PS**	-		
	**EP**	0.121	-	
	**HS**	0.488	0.607	-
	**ES**	1.006	0.935	0.346

The effect size was classified as d(0.01) = very small, d(0.2) = small, d(0.5) = medium, d(0.8) = large, d(1.2) = very large, and d(2.0) = huge. CMJ: height attained in the vertical countermovement jump (cm); T20: time in the 20 m running sprint; V-CUT: time in the V-CUT test, which was conducted to assess the change of direction performance; V1-LOAD: absolute load (kg) lifted at a mean propulsive velocity of 1.00 m·s^−1^ in the full-squat exercise.

**Table 4:** Relationships between the different high-intensity performance indicators assessed in female football players (n = 27).

	T20	V-CUT	V1-LOAD
*CMJ*	−0.66 ***	−0.18	0.03
*T20*		0.32	0.13
*V-CUT*			−0.39 *

CMJ: height attained in the vertical countermovement jump (cm); T20: time in the 20 m running sprint; V-CUT: time in the V-CUT test, which was conducted to assess the change of direction performance; V1-LOAD: absolute load (kg) lifted at a mean propulsive velocity of 1.00 m·s^−1^ in the full-squat exercise. Significant relationships are indicated as * *p* < 0.05 and *** *p* < 0.001.

## Data Availability

The data that support the findings of this study are available on request from the first author.
